# Supplementary material for: The Effects of Cancer Beliefs and Sociodemographic Factors on Colorectal Cancer Screening Behaviours in Newfoundland and Labrador
Source: Healthcare (Basel). 2022 Dec 19;10(12):2574. doi: 10.3390/healthcare10122574 (PMC9778754; doi:10.3390/healthcare10122574)
Supplement: Supplementary file 1 [file healthcare-10-02574-s001.zip › healthcare-2013538-supplementary.pdf]

Supplementary File S1. Survey Items

This is supplemental file to the manuscript entitled "The effects of cancer beliefs and sociodemographic factors on colorectal cancer screening behaviours in Newfoundland and Labrador" by Kong et al., published in the *Healthcare*.

Several items (C1, C2, C3, C4, D1a, D1b, and D1c) in this instrument were adapted from the *Awareness and Beliefs about Cancer (ABC) Instrument* developed by Simon et al. (2011) in collaboration with the International Cancer Benchmarking Partnership and the Cancer Research UK Health Behaviour Research Center. We would also like to acknowledge the work of Stubbings et al. (2009) and the Cancer Research UK Health Behaviour Research Center in developing the *Cancer Awareness Measure (CAM),* as many of the questions in the *ABC Instrument* were developed initially for the *CAM Instrument.* The items in this supplemental file were reproduced with permission.

The footnotes in this file provide additional information on how certain items were scored and on any major changes which were made from the original instruments.

Prevention of Cancer with Lifestyle Modifications: bridging research, health awareness, and practice in Newfoundland and Labrador

CONSENT AND ELIGIBILITY

Do you consent to participating in this study?
By selecting “Yes” and click "Next," you are consenting to be in this study. It tells us you understand the information about the research study. When you select “yes” and consent to be a participant in this study, you are not giving up your legal rights. Researchers or agencies involved in this research study still have their legal and professional liabilities

- Yes
- No

Eligibility Question 1. Are you between 35 and 74 years old?

- Yes
- No

Eligibility Question 2. Are you a resident of Newfoundland & Labrador and have lived here for at least the past 2 years?

- Yes
- No

SECTION A. *General Physical and Social Well-Being*

We would like to start by asking you a few questions about your health.

A1. **Would you say that your general health is:** *(select one)*

5=• Excellent
4=• Very Good
3=• Good
2=• Fair
1=• Poor

A2. **Thinking about the amount of stress in your life, would you say that most of your days are:** *(select one)*

5=• Not at all stressful
4=• Not very stressful
3=• A bit stressful
2=• Quite a bit stressful
1=• Extremely stressful

A3. **Please list any “long-term” chronic health conditions that you have been diagnosed with by a health professional**: *(Long-term means they have lasted or are expected to*

last at least 6 months or more).[1]

____

A4. **Have you ever been diagnosed with cancer?**

2=• Yes
1=• No

A5. **We would like to know if any of your friends or family members have ever been diagnosed with cancer:**

		Yes	No	Not sure
5a.	A significant other (e.g. husband/wife, partner, etc.)	• =2	• =1	• =9
5b.	A close family member (e.g. parents, siblings, children, etc.)	• =2	• =1	• =9
5c.	Other family member (e.g. aunt/uncle, grandparents, cousins, etc.)	• =2	• =1	• =9
5d.	A close friend	• =2	• =1	• =9
5e.	An acquaintance	• =2	• =1	• =9

SECTION B. *Health Care*

B1. **Do you have a regular health care provider? By this, we mean one health professional that you regularly see or talk to when you need care or advice for your health?**

2=• Yes
1=• No

Medical Screening History

There are a variety of screening tests to look for different cancers. Some background information on the a few of the lesser-known tests include the following: FIT, FOBT, colonoscopy, flexible sigmoidoscopy.

*FIT (fecal immunochemical test) or FOBT (fecal occult blood test) check for blood in your stool, where you have to use a stick to smear a small sample of fecal matter onto a special card that gets sent into the lab

‡A colonoscopy is a test where a tube is inserted into the rectum to view the entire bowel, and usually requires anesthetic, whereas a ‡flexible sigmoidoscopy is a shorter tube that only examines the distal part of the colon.

[1] We counted the number of conditions listed and entered this as a number. We then converted this into a categorical variable: none (=0), one to two (=1), 3 or more (=2)

B2. How many years ago did you last have a FIT or FOBT blood screening test for Colorectal Cancer? *(Select from drop-down menu)*[2]

0=• Never had one
1=• Less than 1 year ago
2=• 1 year ago
3=• 2 years ago
4=• 3 years ago
5=• 4 years ago
6=• 5 years ago
7=• 6 years ago
8=• 7 to 10 years ago
9=• 11 or more years ago

B3. How many years ago did you last have a flexible sigmoidoscopy screening test for Colorectal Cancer? *(Select from drop-down menu)*

0=• Never had one
1=• Less than 1 year ago
2=• 1 year ago
3=• 2 years ago
4=• 3 years ago
5=• 4 years ago
6=• 5 years ago
7=• 6 years ago
8=• 7 to 10 years ago
9=• 11 or more years ago

[2] For Questions B2 through B3, we recoded 0 as "0" (Never had screening), and 1 through 9 as "1" (Ever had screening). If an individual had any one of the two screening tests, or both, they were classified as having had CRC screening. Individuals who responded "Never had one" to both of the questions, or who responded "Never had one" to one of the questions and left the other blank, were classified as "never had any CRC screening." Those who did not respond to either screening question were coded as "system missing." Because colonoscopies are not recommended as a screening modality by the Canadian Task Force on Preventive Health Care, we did not consider having had a colonoscopy as CRC screening.

SECTION C. *Cancer Awareness and Beliefs*

C1. **People hold different beliefs about cancer. What is your opinion about the following common beliefs:** *(check the box that best reflects your agreement with each statement)*

	Beliefs About Cancer (Treatment and Outcomes)	Strongly Disagree	Disagree	Agree	Strongly Agree
1a.	These days, many people with cancer can expect to live normal lives	1=•	2=•	3=•	4=•
1b.	Most cancer treatment is worse than the cancer itself	4=•	3=•	2=•	1=•
1c.	I would not want to know if I have cancer	4=•	3=•	2=•	1=•
1d.	Cancer can often be cured	1=•	2=•	3=•	4=•
1e.	Going to the doctor as quickly as possible after noticing a symptom of cancer could increase chances of surviving	1=•	2=•	3=•	4=•
1f.	Some people think that a diagnosis of cancer is a death sentence. To what extent do you agree or disagree with them?	4=•	3=•	2=•	1=•

SECTION D. *Cancer Screening Awareness and Beliefs*

D1.[3] **People hold different beliefs about cancer screening, and their behaviours influenced by different factors. How much do you agree or disagree with each of the statements below?** *(check the box that best reflects your agreement with each statement)*

		Strongly Disagree	Disagree	Neutral	Agree	Strongly Agree
1a.	I would be so worried about what might be found during screening, that I would prefer not to do it.	5=•	4=•	3=•	2=•	1=•
1b.	Cancer screening is only necessary if I have symptoms.	5=•	4=•	3=•	2=•	1=•
1c.	Cancer screening could reduce my chances of dying from cancer.	1=•	2=•	3=•	4=•	5=•
1d.	If I have a healthy lifestyle, I don’t need to worry about having regular cancer screening	5=•	4=•	3=•	2=•	1=•
1e.	Cancer screenings are now very routine tests	1=•	2=•	3=•	4=•	5=•
1f.	Cancer screening tests have a high risk of leading to unnecessary surgery	5=•	4=•	3=•	2=•	1=•

[3] The original ABC (Awareness and Beliefs about Cancer) instrument used a different scoring scale (“Strongly Agree”; “Tend to Agree”; “Tend to Disagree”; and “Strongly Disagree”). Only items D1a-c were from the ABC instrument. Items D1e-h were adapted from the following source under their Creative Commons Attribution License which allowed unrestricted use and reproduction with appropriate citation: Beydoun, H. A., Khanal, S., Beydoun, M. A., Zonderman, A. B., Mohan, R., & Parks-Savage, A. (2014). Are symptoms of anxiety and depression associated with colorectal screening perceptions and behaviors among older adults in primary care? Open Journal of Preventive Medicine, 04(02), 78–89. https://doi.org/10.4236/ojpm.2014.42012.

1g.	Regular cancer screening would give me a feeling of control over my health	1=•	2=•	3=•	4=•	5=•
1h.	I would be more likely to participate in screening if my doctor told me how important it was	1=•	2=•	3=•	4=•	5=•

SECTION H. *Demographic Information*

H1. **What gender do you identify as?**
1=• Female 2=• Male 9=• Other user-missing=• Prefer not to say

H2. **In what age category do you belong?** *(select one)*
1=• 35 – 39 2=• 40 – 44 3=• 45 – 49 4=• 50 – 54
5=• 55 – 59 6=• 60 – 64 7=• 65 – 69 8=• 70 – 74

H3. **Which best describes your ethnic group?** 1=• Caucasian/white 2=• Other

H4. **What is your body weight?** (please specify kilograms or pounds)
For example, if you weigh 165 pounds, please write either "165 pounds" OR "74.8 kilograms[4]

____

H5. **What is your body height?** (please specify centimetres or feet and inches)
For example, if you are 5 feet 11 inches, write "5 feet 11 inches" OR "180 centimetres"

____

H6. **What are the first three characters of your residential (home) postal code?**[5] ______

H8. **Are you currently living with a partner or significant other (such as a husband/wife, common-law partner, or romantic other)?** *(select one)*
1=• No, I am not living with a partner or significant other
2=• Yes, I am living with a partner or significant other

H9. **What is the highest level of education you have completed?**[6] *(select one)*
1=• Did not complete high school
2=• High school
3=• College diploma or university degree
4=• Graduate, Postgraduate, or Professional Degree

[4] Height and weight were used to calculate BMI
[5] Postal codes with a '0' as the second character, based on Canada Post's definition of rural, were assigned as "Rural"(=1) and all other postal codes were considered "Urban"(=2)
[6] We recoded 0 through 1 as "Low Education" (=1) and 3 through 4 as "High Education" (=2)

H10. Which of the following categories best describes your total annual household income before taxes? *(select one)*[7]

1=• Less than $12,000	3=• $60,000 to $79,999
1=• $12,000 to $29,999	3=• $80,000 to $99,999
2=• $30,000 to $49,999	3=• More than $100,000
2=• $50,000 to $59,999	9=• Don't know

This is the end of the questionnaire. Thank you very much for participating.

---

[7] Response categories for income were combined into three categories: <$30,000 "Low Income" (=1), $30,000-59,999 "Middle Income" (=2), and $60,000+ "High Income" (=3)
